# Natural Products, Alone or in Combination with FDA-Approved Drugs, to Treat COVID-19 and Lung Cancer

**DOI:** 10.3390/biomedicines9060689

**Published:** 2021-06-18

**Authors:** Liyan Yang, Zhonglei Wang

**Affiliations:** 1 School of Physics and Physical Engineering, Qufu Normal University, Qufu 273165, China; yangly@iccas.ac.cn; 2 Key Laboratory of Green Natural Products and Pharmaceutical Intermediates in Colleges and Universities of Shandong Province, School of Chemistry and Chemical Engineering, Qufu Normal University, Qufu 273165, China; 3 Key Laboratory of Life-Organic Analysis of Shandong Province, School of Chemistry and Chemical Engineering, Qufu Normal University, Qufu 273165, China; 4 School of Pharmaceutical Sciences, Tsinghua University, Beijing 100084, China

**Keywords:** natural product, SARS-CoV-2, lung cancer, United States Food and Drug Administration-approved drug, natural remedy

## Abstract

As a public health emergency of international concern, the highly contagious coronavirus disease 2019 (COVID-19) pandemic has been identified as a severe threat to the lives of billions of individuals. Lung cancer, a malignant tumor with the highest mortality rate, has brought significant challenges to both human health and economic development. Natural products may play a pivotal role in treating lung diseases. We reviewed published studies relating to natural products, used alone or in combination with US Food and Drug Administration-approved drugs, active against severe acute respiratory syndrome coronavirus 2 (SARS-CoV-2) and lung cancer from 1 January 2020 to 31 May 2021. A wide range of natural products can be considered promising anti-COVID-19 or anti-lung cancer agents have gained widespread attention, including natural products as monotherapy for the treatment of SARS-CoV-2 (ginkgolic acid, shiraiachrome A, resveratrol, and baicalein) or lung cancer (daurisoline, graveospene A, deguelin, and erianin) or in combination with FDA-approved anti-SARS-CoV-2 agents (cepharanthine plus nelfinavir, linoleic acid plus remdesivir) and anti-lung cancer agents (curcumin and cisplatin, celastrol and gefitinib). Natural products have demonstrated potential value and with the assistance of nanotechnology, combination drug therapies, and the codrug strategy, this “natural remedy” could serve as a starting point for further drug development in treating these lung diseases.

## 1. Introduction

As a traditional source for modern pharmaceutical discovery and potential drug leads, natural products have played an integral role in treating patients due to their unique structural, chemical, and biological diversity [1,2,3]. The current race to identify efficacious drugs, natural products with promising therapeutic effects has attracted significant attention, especially for the prevention and treatment of lung diseases, such as pulmonary fibrosis [4], asthma [5], acute lung injury [6], chronic obstructive pulmonary disease [7], defective pulmonary innate immunity [8], coronavirus disease 2019 (COVID-19) [9], and lung cancer [10]. Among the myriad of known lung maladies, COVID-19 and lung cancer are currently the most important public health concerns and burdens worldwide [11,12].

The highly contagious COVID-19 pandemic, caused by severe acute respiratory syndrome coronavirus 2 (SARS-CoV-2), has spread quickly across all continents [13,14]. Presently, this global pandemic has posed a significant threat to the lives of billions of individuals through human-to-human transmission [15,16]. In this scenario, the rapid discovery of efficacious agents against the fast-spreading COVID-19 pandemic is currently a top priority of research across the world [17]. Lung cancer, globally, is a malignant tumor with the highest mortality rate (accounting for 18% of all cancer deaths), and the five-year survival rate is very low (only 10% to 20%) [18]. Non-small cell lung cancer (NSCLC), a subtype of lung cancer with the highest incidence rate (accounting for about 85% of lung cancer [19]), has brought significant threats and challenges to human life and health as well as social and economic development. In this context, more aggressive drug trial protocols investigating anti-lung cancer agents are another top research priority [20].

Significant progress had been made in the understanding of natural products active against COVID-19 and lung cancer. However, there has been no hierarchical review (natural product, monotherapy, or in combination with a US Food and Drug Administration (FDA)-approved drug) covering the use of natural products (including natural product-based nanoparticles) as high-quality therapeutic agents for the treatment of COVID-19 or lung cancer in the literature. To underline systematically the potential importance of natural products, including their biological activity and underlying molecular mechanisms, this review will focus on the current knowledge of potential anti-COVID-19 or anti-lung cancer agents. To explore the therapeutic value of natural products better, we have focused on the current progress in representative chemical components against SARS-CoV-2 and lung cancer based on evidence from promising in vitro studies published from 1 January 2020 to 31 May 2021 by interrogating online databases (such as Google Scholar, ACS Publications, Wiley, MDPI, Web of Science, Science Direct, Springer, PubMed, and X-MOL), rather than taking an exhaustively literature-driven approach. Our purpose is to provide a promising “natural remedy” for the treatment of lung cancer and COVID-19.

## 2. Natural Products as Monotherapy for the Treatment of SARS-CoV-2

**Figure 1:**
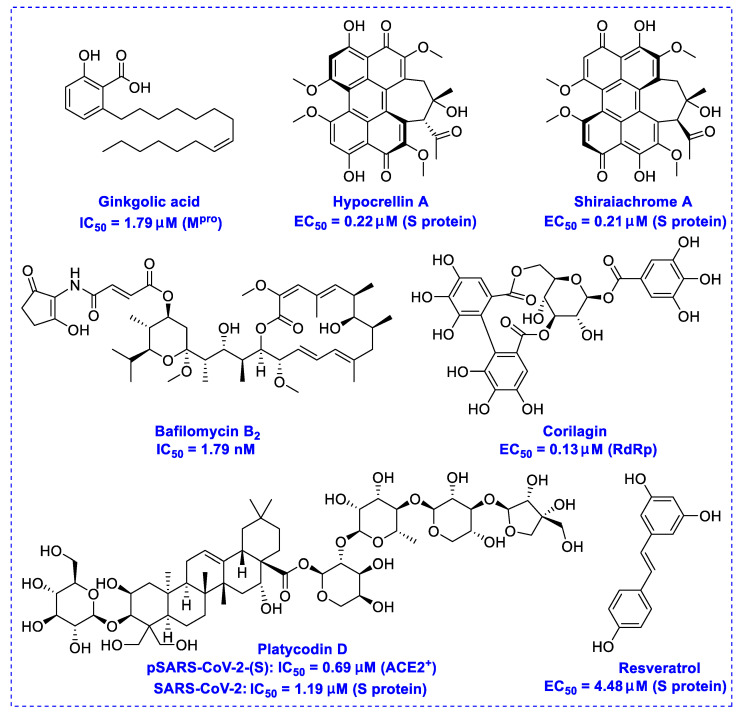
Promising natural products for treating SARS-CoV-2.

Natural products have demonstrated potential value, which supports this strategy as an indispensable research focus in the fight against the COVID-19 epidemic [21,22]. The chemical structures of the components described in this section are shown in Figure 1. The SARS-CoV-2 main protease (M^pro^), also called the 3C-like protease (3CL^pro^), has a vital function in viral replication and is, therefore, a preferred drug target [23]. The papain-like protease (PL^pro^), another prime therapeutic target, plays an essential role in maturing viral RNA polyproteins and dysregulation of host inflammation [24]. Ginkgolic acid, a phenolic acid, is an essential component of the traditional herbal medicine *Ginkgo biloba* (EGb) [25]. A study has demonstrated that ginkgolic acid is characterized by half-maximal inhibitory concentration (IC_50_) values of 1.79 μM and 16.3 μM against SARS-CoV-2 M^pro^ and SARS-CoV-2 PL^pro^, respectively [26]. The study unambiguously showed that ginkgolic acid exerts good dual-inhibitory effects through its irreversible binding to SARS-CoV-2 cysteine proteases [26].

Angiotensin-converting enzyme 2 (ACE2), an essential ingredient of the renin–angiotensin–aldosterone system (RAAS), is a critical host cell surface receptor for viral infection [27]. The glycosylated spike protein (S protein) plays an essential role in mediating viral entry via interactions with the ACE2 cell surface receptor [28]. Hypocrellin A and shiraiachrome A, two-axial chiral perylenequinones, have been reported to exhibit potent effects on the infected monkey Vero E6 cell line by inhibiting the activity of the SARS-CoV-2 S protein at EC_50_ values of 0.22 μM and 0.21 μM, respectively, while at doses of up to 10 μM, these presented no observable cytotoxicity against these cells [29].

Transmembrane protease serine 2 (TMPRSS2), a critical factor enabling SARS-CoV-2 infection, can interact with ACE2 [30]. It has been reported that platycodin D, a triterpenoid saponin isolated from *Platycodon grandiflorum*, prevents TMPRSS2-driven infection in vitro by impairing membrane fusion [31]. Platycodin D has IC_50_ values of 0.69 μM and 0.72 μM for SARS-CoV-2 pseudovirus (pSARS-CoV-2) overexpression of ACE2 (ACE2^+^) and ACE2/TMPRSS2^+^, respectively, and IC_50_ values of 1.19 μM and 4.76 μM for SARS-CoV-2 in TMPRSS2-negative Vero cells and TMPRSS2-positive Calu-3 cells, respectively [31]. Resveratrol, a remarkable phytoalexin, may effectively inhibit the replication of SARS-CoV-2 S protein in Vero E6 cells at an EC_50_ of 4.48 μM [32], and has an excellent safety tracking record, with no cytotoxicity even up to a concentration of 150 µM [33].

The RNA-dependent RNA polymerase (RdRp) of SARS-CoV-2 is another promising target that regulates the replication of the viral genome [34]. Corilagin, a non-nucleoside inhibitor, is a gallotannin isolated from the medicinal plant *Phmllanthi Fructus* [35]. Corilagin has been reported to inhibit SARS-CoV-2 infection with an EC_50_ value of 0.13 μM in a concentration-dependent manner by preventing the conformational change of RdRp and inhibits SARS-CoV-2 replication [36]. Furthermore, corilagin, as identified via molecular dynamics simulation-guided studies, could also be used as an endogenous M^pro^ candidate, with an 88% anti-SARS-CoV-2 M^pro^ activity at concentrations of 20 μM in vitro [37].

Bafilomycin B_2_, which can be isolated from *Streptomyces* sp. HTL16, indicates enhanced inhibitory potency against SARS-CoV-2 at IC_50_ values of 5.11 nM (in the full-time approach) and 8.32 nM (in the pretreatment-of-virus approach) in Vero E6 cells, respectively [38]. While bafilomycin B_2_ has demonstrated potential effectiveness in inhibiting the viral entry process, evidence of its utility as anti-SARS-CoV-2 agents in vivo is currently insufficient.

The above evidence supports the potential value of the above natural products as therapeutic agents for the treatment of the novel SARS-CoV-2 infection, suggesting more validation studies (both in vitro and in animal models as well as on humans) could be encouraged to perform. Besides the above-mentioned molecules, several other natural products have also been shown to exhibit potent anti-SARS-CoV-2 activities in vitro. Table 1 summarizes a range of studies investigating the in vitro effects of anti-SARS-CoV-2 agents since 2020.

Traditional Chinese medicines have attracted considerable attention due to their ability to effectively inhibit SARS-CoV-2 [63,64,65]. For example, the Qingfei Paidu decoction (QFPD) has shown an ability to treat COVID-19 patients at all stages with excellent clinical efficacy (cure rate >90%) [66,67]. Shuanghuanglian oral liquid or injection (SHL), another well-known traditional Chinese medicine, dose-dependently inhibits SARS-CoV-2 M^pro^ replication [68]. In addition to the above-mentioned QFPD and SHL, several other traditional Chinese medicines (such as Kegan Liyan oral liquid and Toujie Quwen granule) listed in Table 2 contain *Scutellaria baicalensis* Georgi (Chinese name: Huangqin), whose major component is baicalein, exerts a marked anti-SARS-CoV-2 effect (IC_50_ of 0.94 μM, and SI > 212) [69]. Furthermore, it is crucial to investigate how herbal medicine affects SARS-CoV-2 infection by studying its active ingredients. To elucidate the underlying molecular mechanisms, a crystal structure of SARS-CoV-2 M^pro^ complexed with baicalein was constructed at a resolution of 2.2 Å (the Protein Data Bank (PDB) ID: 6M2N) [68]. Analysis of the core of the substrate-binding pocket revealed multiple interactions (such as hydrogen bonding with Leu141/Gly143 and Ser144/His163, π–π interactions with Cys145 and His4, and hydrophobic interactions with Met49 and His41), which effectively blocked SARS-CoV-2 replication via noncovalent incorporation [68]. The relevant studies [70,71,72] provided direct data for a better understanding of the molecular mechanisms of Chinese herbal medicine by studying its active ingredients.

## 3. Natural Products as Monotherapy for the Treatment of Lung Cancer

**Figure 2:**
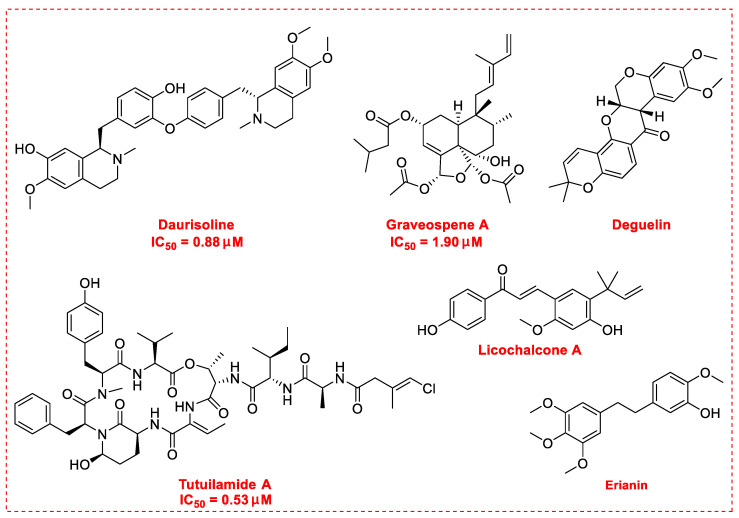
Promising natural products for treating lung cancer.

There is no doubt that natural products have always been recognized as promising anti-lung cancer agents. Daurisoline, an autophagy blocker, is a bisbenzylisoquinoline alkaloid extracted from the herbal medicine *Nelumbo nucifera* Gaertn [74]. The chemical structures of the molecules discussed in this section are shown in Figure 2. Daurisoline increases the degradation of β-catenin by targeting heat shock protein 90 (HSP90) directly and decreases the expression of MYC proto-oncogene (c-MYC) and cyclin D1, which resulted in cell cycle arrest at the G1 phase in human lung cancer A549 cells and Hop62 cells lines to exert its anti-lung cancer activity [75]. More importantly, in animals, daurisoline has been reported to be a promising anti-lung cancer agent (by inhibiting tumor growth in lung cancer xenografts) with no observable side effects, thus highlighting a potential role for daurisoline in the treatment of lung cancer [75]. Another recent study has shown that daurisoline can effectively inhibit SARS-CoV-2 replication at IC_50_ values of 3.664 μM and 0.875 μM in Vero E6 cells and in human pulmonary alveolar epithelial cells (HPAEpiC), respectively [49].

Graveospene A, isolated from the leaves of *Casearia graveolens*, is a new clerodane diterpenoid that has been reported to induce apoptosis in A549 cells with an IC_50_ value of 1.9 μM by inducing cell cycle arrest in phase G0/G1 [76]. Deguelin, a protein kinase B (AKT) kinase inhibitor, is isolated from the African plant *Mundulea sericea* (Leguminosae) and is commonly used to inhibit the growth of several types of human cancer cell lines [77]. Deguelin promoted the phosphorylation of myeloid cell leukemia sequence-1 (Mcl-1) protein and induced the inhibition of the wildtype and mutated epidermal growth factor receptor (EGFR)-Akt signaling pathway, which resulted in activation of downstream GSK3β/FBW7 and profound anti-NSCLC activity with no obvious side effects in vivo [78].

Licochalcone A is a natural flavonoid derived from *Xinjiang licorice* and *Glycyrrhiza inflata*. Licochalcone A is known to possess a broad spectrum of activities with important pharmacological effects in various cancer cell lines [79]. Licochalcone A can significantly increase autophagic cytotoxicity (in both A549 and H460 cell lines) and downregulated the expression of c-IAP1, c-IAP2, XIAP, survivin, c-FLIPL, and RIP1, apoptosis-related proteins via inhibiting the activity of phosphorylated extracellular signal-regulated kinase (ERK) and autophagy [80]. In addition, licochalcone A has been reported to abolish the expression of programmed death ligand-1 (PD-L1) by increasing reactive oxygen species (ROS) levels in a time-dependent manner and interfering with protein translation in cancer cells [81]. Further, licochalcone A can inhibit PD-L1 translation likely through the inhibition of the phosphorylation of 4EBP1 and activation of the PERK-eIF2α signaling pathway [81]. Licochalcone A plays a vital role in reversing the ectopic expression of key microRNA (miR-144-3p, miR-20a-5p, miR-29c-3p, let-7d-3p, and miR-328-3p) to elicit lung cancer chemopreventive activities both in vivo and in vitro [82]. In addition, licochalcone A has been reported to inhibit EGFR signaling and reduced the expression of Survivin protein in a cap-dependent translation manner to exhibit profound activity in mutated NSCLC cells [83].

Erianin, a novel dibenzyl compound, can be isolated from the traditional herbal medicine *Dendrobium chrysotoxum* Lindl and has been proposed as an apoptosis-inducing agent in human lung cancer cells [84]. The main mechanisms of its anti-lung cancer activity involve the induction of ferroptosis by activating Ca^2+^/calmodulin signaling, inhibition of cell proliferation and metastasis, and induction of cell cycle arrest in phase G2/M [85].

Tutuilamide A, isolated from marine cyanobacteria *Schizothrix* sp., is a novel cyclic peptide reported to exhibit moderate cytotoxicity activity in the H-460 human lung cancer cell line with an IC_50_ value of 0.53 μM [86]. Tutuilamide A, with the help of the vinyl chloride side chain, showed enhanced inhibitory potency with high selectivity (IC_50_ 0.73 nM) for human neutrophil elastase, which is associated mainly with the migration and metastasis of lung cancer cells [87]. Besides the above-mentioned molecules, Table 3 also exhibits other natural products (including their underlying molecular mechanisms) with notable anti-lung cancer activities reported since 2020.

## 4. Natural Products in Combination with the FDA-Approved Drugs Inhibit SARS-CoV-2

The bisbenzylisoquinoline alkaloid cepharanthine can be isolated from the traditional herbal medicine *Stephania cephalantha* Hayata [132]. Cepharanthine exhibits a range of promising bioactivity. It has IC_50_ values of 0.026 μM, 9.5 μg/mL, and 0.83 μM against the human immunodeficiency virus type 1 (HIV-1) [133], SARS-CoV [134], and human coronavirus OC43 (HCoV-OC43) [135], respectively. This alkaloid inhibits SARS-CoV-2 entry in vitro at an IC_50_ of 0.35 μM without any evident toxicity profile (selectivity index, [SI] > 70) [136]. Furthermore, the cell death cascade induced by the cellular stress response is another key target for SARS-CoV-2 [137]. It is worth noting that this bisbenzylisoquinoline alkaloid, with a good safety profile, is an approved drug in Japan since the 1950s and is used to treat acute and chronic diseases [132], highlighting that cepharanthine can serve as a potential therapeutic candidate for the treatment of SARS-CoV-2 infection.

Nelfinavir (Viracept), the first HIV-1 protease inhibitor developed by Agouron Pharmaceuticals, was approved by the FDA in March 1997 for the treatment of HIV-AIDS [138]. Recently, nelfinavir was shown to be effective at inhibiting SARS-CoV-2 M^pro^ infection (IC_50_ = 3.3 μM) with a low level of toxicity (SI = 3.7) [139]. In addition, nelfinavir inhibited SARS-CoV-2 replication in vitro with an EC_50_ of 1.13 μM [140]. Nelfinavir was also effective at dose-dependently inhibiting SARS-CoV-2 S protein—complete inhibition at the concentration of 10 μM—with no evidence of cellular cytotoxicity [141]. Remarkably, nelfinavir can also improve lung pathology caused by SARS-CoV-2 infection [142]. Nonetheless, nelfinavir might not benefit SARS-CoV-2-infected patients by reducing viral loads in the lungs, just as it does not reduce viral load in hamsters [142].

**Figure 3:**
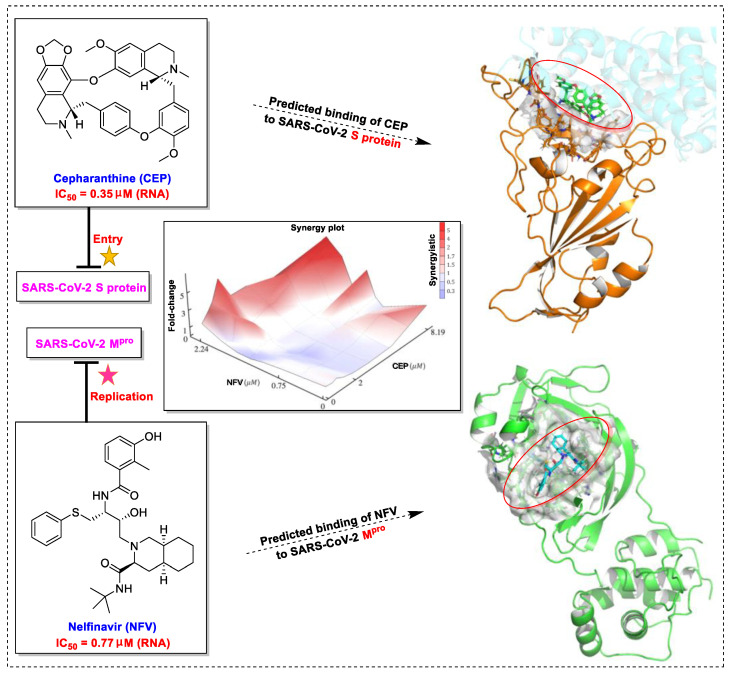
Cepharanthine in combination with FDA-approved nelfinavir inhibits SARS-CoV-2.

Taken together, numerous studies have demonstrated the in vitro anti-SARS-CoV-2 activity of cepharanthine (via inhibition of SARS-CoV-2 S protein) and nelfinavir (via inhibition SARS-CoV-2 M^pro^ and partly S protein). To reveal the synergistic efficacy (Figure 3) of the above two molecules in SARS-CoV-2 infected patients, based on models of pharmacokinetics, pharmacodynamics, and viral-dynamics, Ohashi et al. constructed a mathematical prediction model of the therapeutic effects and revealed that the combination of cepharanthine (intravenous) and nelfinavir (oral) showed excellent synergistic effects in COVID-19 patients (with viral clearance occurring 1.23 days earlier than with nelfinavir alone; cepharanthine alone showed a minimal effect) [136]. Considering all these factors, including the critical value of cepharanthine and nelfinavir in anti-SARS-CoV-2 infection, both in vitro and in animal models and mathematical prediction modeling, further research is needed to explore whether these molecules exert synergistically augmented activity for the treatment of SARS-CoV-2 infection in patients. It is worth noting that further research is needed to explore whether they have anti-SARS-CoV-2 activity in vivo.

Remdesivir (GS-5734, Veklury^®^), an RdRp inhibitor developed by Gilead Science, was the first, and currently the only, anti-SARS-CoV-2 drug approved by the FDA (approval on 22 October 2020) for the treatment of COVID-19 [143,144,145]. Remdesivir exhibits broad-spectrum activity against multiple viral infections in vitro, including SARS-CoV, Middle East respiratory syndrome coronavirus (MERS-CoV), Ebola virus (EBOV), and SARS-CoV-2, with EC_50_ values of 0.069 μM, 0.090 μM, 0.012 μM, and 0.77 μM, respectively [146,147,148,149]. Furthermore, remdesivir has also been thoroughly explored in animal models. Remdesivir reduced lung viral loads in MERS-CoV-infected rhesus monkeys [150] and transgenic *Ces1c*^−^/^−^ *hDPP4* mice [147], protected Nipah virus-infected African green monkeys [151] and rhesus macaques from SARS-CoV-2 infection [152]. Moreover, since 2016, the efficacy and safety of remdesivir have been clinically investigated for the treatment of EBOV infection [153]. Nonetheless, the FDA-approved remdesivir does not appear highly effective in the fight against the COVID-19 pandemic [154,155,156]. In this scenario, the combination of remdesivir with other small molecules, including natural products and natural-product-inspired potential anti-SARS-CoV-2 agents, may exhibit a synergistic effect, compared to remdesivir alone in COVID-19 patients.

Linoleic acid, an inflammatory response modulator [157] isolated from the traditional meal *Vicia faba* [158], significantly suppresses MERS-CoV replication [159]. Toelzer et al. hypothesized that the combination of remdesivir and linoleic acid, an essential diunsaturated fatty acid, may be superior for treating COVID-19 patients over remdesivir alone [160]. Indeed, the combination of linoleic acid (50 μM) and remdesivir (20 to 200 nM) exerted a synergistic effect on SARS-CoV-2 replication in human Caco-2 ACE2+ cells in vitro [160].

**Figure 4:**
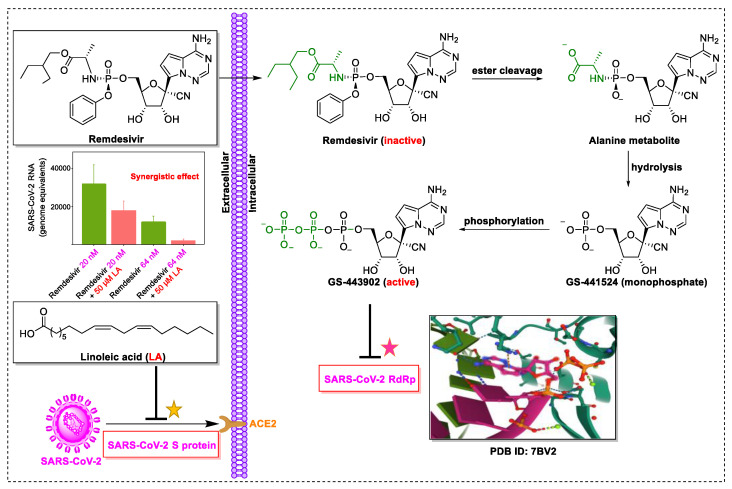
Linoleic acid in combination with FDA-approved remdesivir inhibit SARS-CoV-2.

The synergistic mechanisms involved in the combination of linoleic acid and remdesivir shown in Figure 4. To clarify the underlying inhibitory mechanisms of action of linoleic acid, a cryo-electron microscopy (cryo-EM) model of SARS-CoV-2 S protein complexed with linoleic acid was determined at 2.85 Å resolution (Electron Microscopy Data (EMD) ID: 11145) [160]. Further analysis of the linoleic acid binding pocket within the S protein revealed that the hydrocarbon tail of linoleic acid binds to hydrophobic amino acids. At the same time, the acidic headgroup interacts with a positively charged anchor (Arg408 and Gln409) to lock the S protein irreversibly. The hydrophobic pocket with a tube-like shape of the S protein allows a good fit for linoleic acid, and results in reduced ACE2 interactions, and thus sets the stage for an intervention strategy that targets linoleic acid binding to SARS-CoV-2 S protein [160].

As for remdesivir, it is a phosphoramidate prodrug, which requires conversion from the parent drug into the active triphosphate form (GS-443902) [161]. In cells, the triphosphate form, GS-443902, can block SARS-CoV-2 replication by evading the “proofreading” activity of viral RNA sequences [162]. In addition, Yin et al. [34] revealed the cryo-EM structure of SARS-CoV-2 RdRp in complex with remdesivir (using its triphosphate metabolite GS-443902) at 2.5 Å resolution (PDB ID: 7BV2) [34]. The cryo-EM structure unambiguously demonstrated that GS-443902 could positioned itself at the center of the catalytic site of the primer RNA, covalently binding to the primer at the 1+ position of the template strand to terminate chain elongation. Three strong H-bonds with active site residues (ribose -OH groups: Asp623, Ser682, and Asn691; sugar 2′-OH: Asn691) were identified [34]. Further research is warranted to establish whether linoleic acid and remdesivir exert synergistic anti-SARS-CoV-2 effects in vivo. At present, a more well-designed combination drug therapy that exhibits better additive or synergistic effects against COVID-19 is a promising strategy. However, for COVID-19, the nanodrug strategy (containing natural products and FDA-approved drugs) remains an open question, and undoubtedly, it has a long way to go.

## 5. Natural Products in Combination with the FDA-Approved Anti-Lung Cancer Drugs

As regards lung cancer, significant progress has been made in the research of natural product-based nanomedicines [163,164] and combination drug therapies [165,166], which can provide some reference for the related drug discovery and development for COVID-19. In this section, we mainly focused on the nanodrug strategy (containing natural products and FDA-approved drugs) to reveal its unique advantage in the research and development of anti-lung cancer drugs.

Curcumin is one of the main products of the *Curcuma longa* L. (turmeric) rhizome extract and has been proposed for its antimicrobial, antimutagenic, antiproliferative, and neuroprotective activities [167]. Curcumin is considered an ideal scaffold for lung cancer drug discovery due to its potent antitumor effects against NSCLC [168]. In particular, several crucial molecular pathways involved in the efficacy of curcumin as an anti-lung cancer drug involve the vascular endothelial growth factor (VEGF), EGFR, nuclear factor-κB (NF-κB), and mammalian target of rapamycin (mTOR) pathways [169]. Nonetheless, the biomedical application of curcumin is currently hindered by its poor aqueous solubility and low bioavailability [170]. In contrast, cisplatin, already marketed as the first platinum-based complex approved by the US FDA, has been used therapeutically for a broad range of cancers such as lung, lymphomas, melanoma, head, and neck cancer [171]. Unfortunately, the routine clinical practice of cisplatin is often coupled with severe toxic side effects (such as nephrotoxicity [172], severe hearing loss [173], and cardiotoxicity [174]) and intrinsic or acquired drug resistance [175].

**Figure 5:**
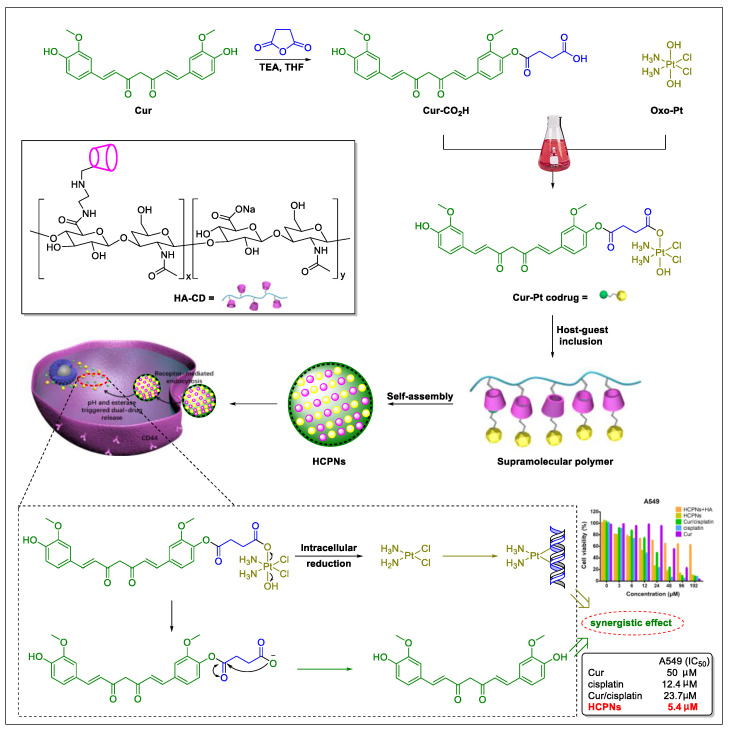
Synthesis, bioconversion, and synergistic effects of the nanoparticles HCPNs (image reproduced with permission from [178].

Indeed, an efficacy study in NSCLC cells evidenced improved effects of the drug combination of curcumin and cisplatin [176]. An in vitro study showed that curcumin enhanced cisplatin-induced therapeutic efficacy in lung cancer cell lines A549, H460, and H1299 by regulating the Cu-Sp1-CTR1 regulatory loop. Furthermore, the promotion of active targeting ability with β-cyclodextrin (β-CD)-modified hyaluronic acid (HA) was identified as an effective strategy to address cellular uptake, intracellular trafficking, and therapy performance of the drug delivery systems [177]. Taking all these factors into account, Bai et al. [178] designed and constructed a β-cyclodextrin-modified hyaluronic acid-based pH- and esterase-dual-responsive supramolecular codrug combining curcumin and cisplatin (Figure 5). In detail, the designed guest moiety Cur-Pt was prepared via esterification reactions between curcumin, oxoplatin, and a molecule of succinic acid. The scheduled host moiety β-CD-modified hyaluronic acid (HA-CD) was prepared via amidation of the carboxylate salt sodium hyaluronate with free amine mono-6-deoxy-6-ethylenediamino-β-CD (β-CD-EDA). Eventually, the desired curcumin and cisplatin nanoparticles (HCPNs) were developed through a host–guest inclusion strategy and subsequent self-assembled. Interestingly, in this targeting system, curcumin acted as both the guest molecule and the chemical anticancer drug.

In vitro evaluation revealed that the HCPNs could be internalized by cancer cells. Once inside the cell, curcumin is released under acidic endosomal conditions (pH-responsive), and cisplatin is released via reducing of oxoplatin under higher expressed glutathione (GSH) conditions (esterase-responsive). Moreover, cell-based experiments revealed the effective cellular toxicity (high efficiency, the IC_50_ value of 5.4 μM in A549 cells) and active targeting ability (low toxicity, with low expression levels in normal LO-2 cells) of this novel drug-delivery system. Given the observed positive synergistic effect in the study, the authors concluded that HCPNs exhibited improved effects, compared with either monotherapy with curcumin or cisplatin [178]. The drug delivery and sustained release behavior of Cur from HCPNs were investigated in vitro at pH 7.4 after 48 h (11% Cur was released) and pH 5.0 after 48 h (79% Cur was released), respectively, proving the better stability than Cur alone [178]. Meanwhile, although the Tian group did not proceed further with their in vivo studies; we suggest additional in vivo studies should be performed to identify the pharmacokinetic or pharmacodynamic profile of the HCPNs and the synergistic activity against lung cancer of this codrug.

The disulfide bond, a promising redox-reactive switch in vivo, plays an essential role in many biological processes [179]. To reduce adverse effects resulting from chemotherapy regimens, the disulfide-based drug design has attracted great enthusiasm in the synthesis of prodrug or codrug, and especially for the preparation of functional nanodrugs due to their high selectivity and biocompatibility [180,181]. The nontoxic nanodrugs are activated by the excess of GSH in the tumor microenvironment, which provides an essential strategy for lung cancer-targeting treatment [182].

Celastrol, a typical pentacyclic triterpenoid, can be extracted from traditional herbal medicines of the Celastraceae family [183]. Celastrol is considered another up-and-coming natural product for lung cancer treatment due to its potent anti-NSCLC activity via its suppression of Axl protein expression [184], initiating tumor necrosis factor-related apoptosis-inducing ligand (TRAIL)-mediated apoptotic cell death [185], and suppressing cell invasion [186]. However, the clinical translation and biomedical application of celastrol are hindered due to its low bioavailability and physiological instability [187].

**Figure 6:**
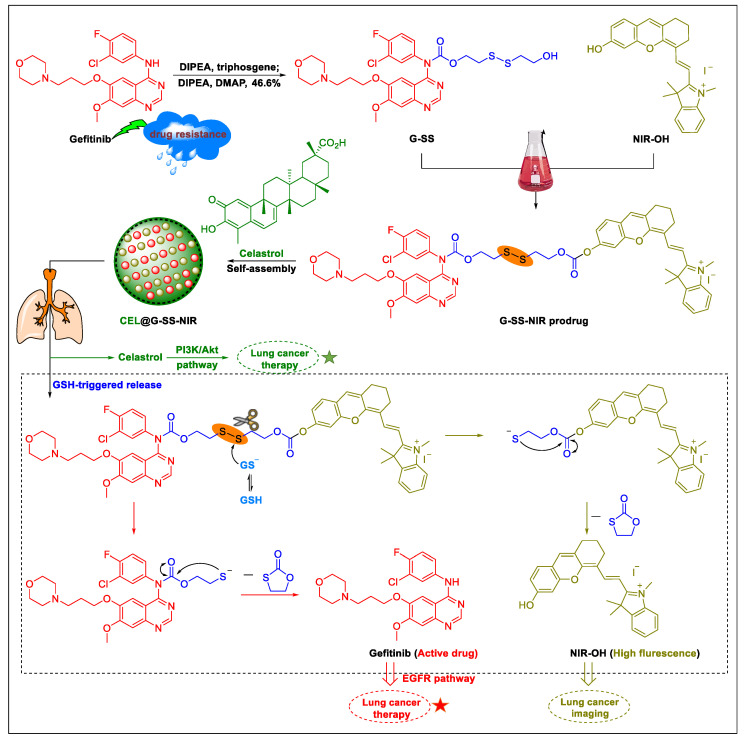
Synthesis, bioconversion, and synergistic effect of the nanodrug CEL@G-SS-NIR.

Gefitinib, approved by US FDA, has been used therapeutically as the first-line agent in patients with advanced lung cancer [188]. Unfortunately, the routine clinical practice of gefitinib is often coupled with severe adverse effects, such as pulmonary toxicity [189], respiratory failure, and severe comorbidities [190]. Following a reasonable design, Wu et al. developed a GSH-responsive nanodrug (identified as CEL@G-SS-NIR in Figure 6), which possesses unique therapeutic efficacy for NSCLC in mice models by inhibiting upstream and downstream EGFR signaling pathways [191]. The nanodrug CEL@G-SS-NIR was prepared in two steps: preparation of the prodrug and acquisition of the nanocomplex. As shown in Figure 6, the main molecule G-SS-NIR of the nanodrug CEL@G-SS-NIR was synthesized through a two-step reaction. First, the key intermediate G-SS was synthesized successfully in the presence of gefitinib (G), 2-hydroxyethyl disulfide (-SS-), and tiphosgene via covalent linkage. Next, the near-infrared (NIR-OH) chromophore was bound to the side chain of the G-SS to form the prodrug G-SS-NIR. The amphiphilic G-SS-NIR readily self-assembled into spherical nanomicelles in an aqueous medium (driven by the disulfide bond and the π–π interaction) and was encapsulated concomitantly the hydrophobic serine-threonine protein kinase (Akt) inhibitor celastrol (marked as CEL) to form CEL@G-SS-NIR.

This novel nanodrug CEL@G-SS-NIR possesses a suitable size (average diameter 119 ± 6 nm), outstanding overall drug loading (64.0 ± 1.4 wt.%), and excellent stability in the blood circulation, and has a rapid release rate of the free molecules (gefitinib, celastrol, and NIR-OH) at tumor region due to the breaking of the disulfide bonds in the presence of high levels of GSH [191].

In vitro, the nanodrug CEL@G-SS-NIR formulation could effectively target the tumor region due to its enhanced permeability and retention effect and also allowed fluorescent imaging in vivo, at a predetermined timepoint after tail vein injection, in orthotopic lung tumors [191]. In the treatment protocol, the mice were randomly divided into five groups (five mice per treatment group), and after a single treatment cycle, the CEL@G-SS-NIR group (13.4 mg/kg, intravenously, for 20 days), compared to the control groups, exhibited stronger NSCLC tumor-suppressive effects [191]. As for the response mechanism involved, the entire process can be divided into four steps: (i) CEL@G-SS-NIR accumulates in the lung tumor region, (ii) CEL@G-SS-NIR releases the drug celastrol and the protonated intermediates (and) through the deprotonated glutathione (GS¯) nucleophilic attack of the disulfide bond on G-SS-NIR bonds, (iii) this further induces the synchronous releases of the parent drug gefitinib and the fluorescent dye NIR-OH via an intramolecular cyclization reaction (thiolate anion moiety reacts with the adjacent carbonyl group), and (iv) finally, the synergistic anticancer activity is activated by suppressing the phosphatidylinositol 3-kinase/serine threonine protein kinase (PI3K/Akt) signaling pathway by celastrol and downregulating EGFR signaling pathway by gefitinib. Simultaneously, a fluorescent and multispectral optoacoustic tomography imaging signal is generated by NIR-OH [192]. This study showed that disulfide-based and targeted fluorescent nano-prodrugs for treating NSCLC and tracking drug delivery systems are particularly advantageous.

## 6. Conclusions and Future Perspectives

COVID-19 and lung cancer, the two most critical lung diseases presenting high mortality rates, have posed a great challenge and a serious threat to human health and economic development. Since 2020, as is well-known, the scientific community has made great efforts and remarkable inroads in developing promising anti-SARS-CoV-2 and anti-lung cancer agents through various approaches. In this scenario, numerous natural products have fueled significant attention and have shown good results as potential therapeutics for the above-mentioned lung diseases. This review highlighted state-of-the-art of important natural products (including their underlying molecular mechanisms), covering studies published between 1 January 2020 and 31 May 2021, in the treatment of the above-mentioned lung diseases. We found that natural products can be applied in vitro as monotherapy for the treatment of SARS-CoV-2 (ginkgolic acid, resveratrol, and baicalein) and lung cancer (graveospene A, deguelin, and erianin), as well as in combination with the FDA-approved drug inhibit SARS-CoV-2 (cepharanthine plus nelfinavir, linoleic acid plus remdesivir) and as codrug formulations with anti-lung cancer activity in vitro (codrug of curcumin and cisplatin). The evidence revealed herein that natural products could serve as a starting point for further drug development both in COVID-19 and lung cancer. It is worth noting, however, that some natural products could be pan-assay interference compounds, which can give false readouts, and close attention should be paid to decrease futile attempts [193,194]. There is currently very little direct data associated with the clinical effect of natural products against SARS-CoV-2 infection. To understand better and explore systematically the activity of natural products, more validation studies, with high-quality evidence (both in vitro and in animal models as well as on humans), are now needed.

To improve the use of natural products, many intensive research efforts (both in vitro and in vivo) are still needed to explore the limitations of these agents, such as poor water solubility, limited oral absorption, low bioavailability, and the poor first-pass effect, which represent the first step to develop promising anti-COVID-19 or anti-lung cancer agents. It is clear that a long way is still ahead for us to realize natural product-based drug discovery and development, as only phase 1–3 clinical trials can ensure that any small molecule inhibitor can be used as a drug.

More aggressive and well-designed combination drug therapies that exhibit better additive or synergistic effects against COVID-19 and lung cancer are a promising strategy. For example, shiraiachrome A exhibits potent effects in Vero E6 cells by inhibiting the activity of the SARS-CoV-2 S protein at EC_50_ values of 0.21 μM; bafilomycin B_2_ presents enhanced inhibitory potency against SARS-CoV-2 at IC_50_ values of 5.11 nM in Vero E6 cells by inhibiting the viral entry process; ginkgolic acid has IC_50_ values of 1.79 μM and 16.3 μM against SARS-CoV-2 M^pro^ and SARS-CoV-2 PL^pro^. Combining the properties of the above-mentioned natural products with FDA-approved drugs (for example, with nelfinavir or remdesivir) could achieve optimal COVID-19 treatment through multitargeted mechanisms of action. In addition, a codrug of a natural product with an FDA-approved drug could achieve a combination booster through multitargeted activity. However, the codrug strategy remains an open question in the treatment of patients with COVID-19. Thus, we suggest researchers pay considerable attention to the development of emerging codrug therapy strategies.

In contrast, precisely fabricated nanodrugs may be a more potent weapon to enhance biocompatibility, minimize toxicity as well as side effects, achieve long-term circulation in the body, as well as sustained release, overcome undesired adverse effects, and expand the modalities of administration (intravenous injection or inhalation). However, for COVID-19, the nanodrug strategy (containing natural products and FDA-approved drugs) remains another open question. Fortunately, significant progress has been made in the research of lung cancer nanomedicines, which can provide some reference for the related drug discovery and development for COVID-19. There is no doubt that there is a long way to go and many difficulties to overcome. Nonetheless, natural products have their advantages. We sincerely hope natural products will be proven a safe and effective “natural remedy” for the treatment of the above-mentioned lung diseases with the assistance of multiple techniques and strategies.

## Figures and Tables

**Table 1:** Other natural products with anti-SARS-CoV-2 activities in vitro.

No.	Name	Structure	EC_50_ or IC_50_ (μM)	Strain	Refs
1	Acetoside	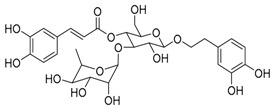	0.043	Vero E6 cells	[39]
2	Anacardic acid	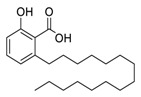	2.07	USA-WA1/2020	[26]
3	Andrographolide	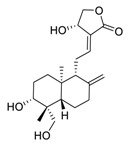	0.034	Calu-3 cells	[40,41]
4	Apigenin-7-*O*-glucoside	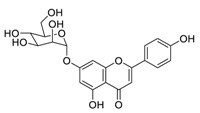	0.074	Vero E6 cells	[39]
5	Artemisinin	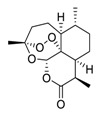	64.45	Vero E6 cells	[41,42]
6	Azithromycin	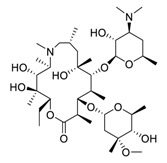	2.12	Caco-2 cells	[43]
7	Baicalin	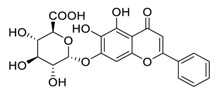	7.98	Vero E6 cells	[44]
8	Cannabidiol	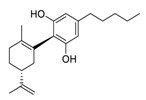	7.91	Vero E6 cells	[45,46]
9	Catechin-3-*O*-gallate	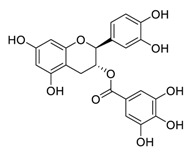	2.98	Vero E6 cells	[47]
10	Chebulagic acid	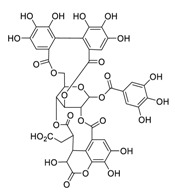	9.76	Vero E6 cells	[48]
11	Daurisoline	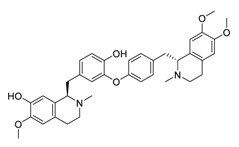	3.66	Vero E6 cells	[49]
12	EGCG	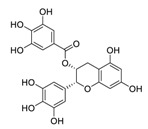	0.874	Vero E6 cells	[44,50]
13	Emetine	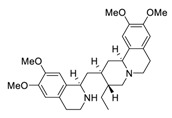	0.46	Vero E6 cells	[51,52]
14	Epicatechin-3-*O*-gallate	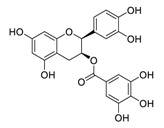	5.21	Vero E6 cells	[47]
15	Gallinamide A	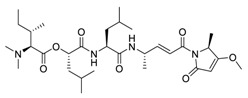	0.028	Vero E6 cells	[53]
16	Gallocatechin-3-*O*-gallate	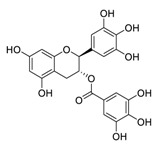	6.38	Vero E6 cells	[47]
17	Hopeaphenol	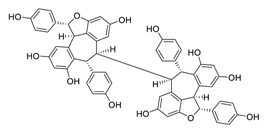	2.3	B.1.351	[54]
18	Ipomoeassin F	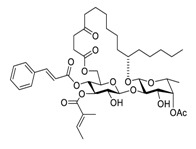		semi-permeabilized mammalian cells	[55]
19	Kobophenol A	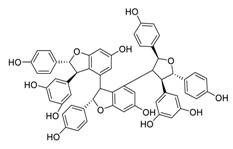	1.81	Vero E6 cells	[56]
20	Myricetin	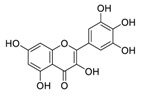	0.22	Vero E6 cells	[57,58]
21	Naringenin	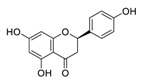	0.092	Vero E6 cells	[39,59]
22	Osajin	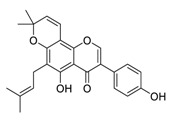	3.87	Vero E6 cells	[60]
23	2,3′,4,5′,6-Pentahydroxybenzophenone	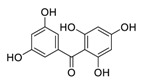	0.102	Vero E6 cells	[39]
24	Procyanidin B2	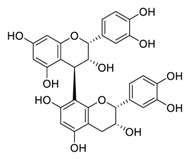	75.3	Vero E6 cells	[47]
25	Punicalagin	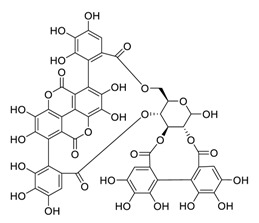	7.20	Vero E6 cells	[48]
26	Sennoside B	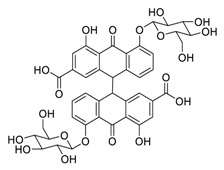	0.104	Vero E6 cells	[39]
27	Shikonin	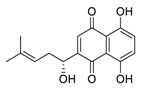	15.75	Vero E6 cells	[61]
28	Δ9-Tetrahydrocannabinol	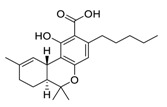	10.25	Vero E6 cells	[45]
29	Tetrandrine	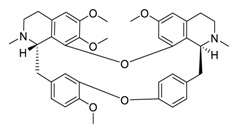	3.00	Vero E6 cells	[60]
30	Theaflavin	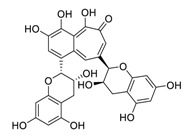	8.44	HEK293T human embryonic kidney cells	[62]

**Table 2:** Registered clinical trials relating to traditional Chinese medicine prescriptions containing baicalein (active ingredient of Huangqin) for treatment of COVID-19 patients (Chinese Clinical Trial Registry, www.chictr.org/cn/ (accessed on 31 January 2021).

Baicalein (The Active Ingredient of Huangqin)	Molecular Mechanisms of Baicalein	Herbal Formula Containing Huangqin	Registration Number	Sample Size of the Control Group
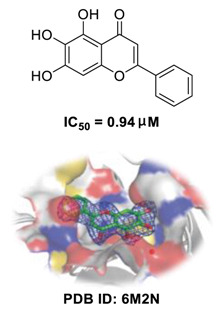	RdRp inhibitor via noncovalent incorporation [73], potent antagonists against TMPRSS2 [70], improving respiratory function, decreasing IL-1β and TNF-α levels, and inhibiting cell infiltration [71,72].	Qingfei Paidu decoction	ChiCTR2000029433	120
			ChiCTR2000030883	100
			ChiCTR2000032767	782
		Xinguan I decoction	ChiCTR2000029637	50
		Tanreqing capsules	ChiCTR2000029813	36
		Tanreqing injection	ChiCTR2000029432	72
		Kegan Liyan oral liquid	ChiCTR2000033720	240
			ChiCTR2000033745	240
			ChiCTR2000031982	240
		Shuanghuanglian oral liquid	ChiCTR2000033133	30
			ChiCTR2000029605	100
		Toujie Quwen granule	ChiCTR2000031888	150

**Table 3:** The mechanism involved in anticancer activities of other natural products (reported since 2020).

No.	Name	Structure	Mechanism of Anti-Lung Cancer	Refs
1	Acovenoside A	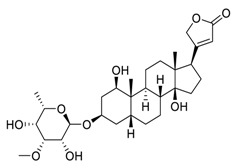	Inhibit the adenosine triphosphate (ATP)-dependent Na+/K+ exchange through the Na+/K+-ATPase	[88]
2	Asiatic acid	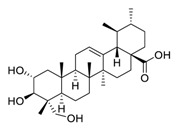	Inhibited the ionizing radiation-induced migration and invasion	[89]
3	Baicalein	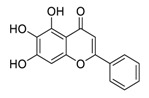	Restrained ezrin tension by decreasing inducible nitric oxide synthase expression levels, suppress invasion, reduced vasculogenic mimicry formation	[90,91,92]
4	Baicalin	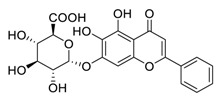	Inhibited the invasion, migration, angiogenesis, and Akt/mTOR pathway	[93,94]
5	Casticin	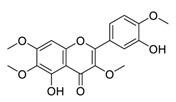	Induced the expressions and nuclear translocation of phosphorylation of H2AX	[95]
6	Dioscin	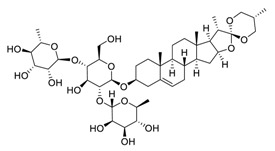	Down-regulated signal transducer and activator of transcription 3 and c-Jun N-terminal kinase signaling pathways	[96]
7	EGCG	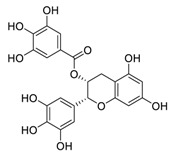	Regulated CTR1 expression through the ERK1/2/NEAT1 signaling pathway	[97,98]
8	Ellagic acid	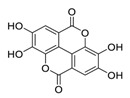	Inhibited tumor growth, increased p-AMPK, and suppressed hypoxia-inducible factor 1α levels	[99]
9	Erianthridin	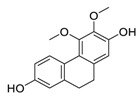	Attenuated extracellular signal-regulated kinase activity and mediated apoptosis, matrix-degrading metalloproteinases (MMPs) expression	[100,101]
10	Eugenol	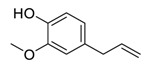	Restriction of β-catenin nuclear transportation	[102]
11	Formononetin	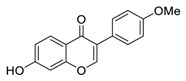	Inhibited EGFR-Akt signaling, which in turn activates GSK3β and promotes Mcl-1 phosphorylation in NSCLC cells	[103,104]
12	Gallic Acid	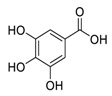	Inhibited of EGFR activation and impairment, inhibition of phosphoinositide 3-kinase (PI3K) and AKT phosphorylation	[105,106]
13	Glochidiol	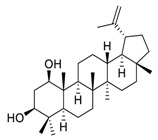	Inhibited tubulin polymerization	[107]
14	Gracillin	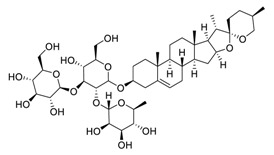	Inhibited both glycolysis and mitochondria-mediated bioenergetics, induced apoptosis through the mitochondrial pathway	[108,109]
15	Hispidulin	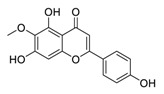	Promoted apoptosis by hispidulin via increased generation of ROS	[110]
16	Icaritin	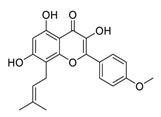	Downregulated the immunosuppressive cytokine (TNF-α, IL10, IL6) and upregulated chemotaxis (CXCL9 and CXCL10)	[111]
17	Isoharringtonine	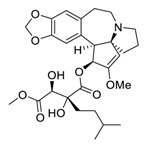	Induced death tumor spheroids by activating the intrinsic apoptosis pathway	[112]
18	Kaempferol	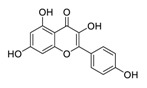	Inhibitor of nuclear factor erythroid 2-related factor 2	[113]
19	Liriopesides B	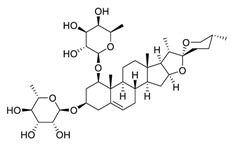	Reduced proliferation, and induced apoptosis and cell cycle arrest, inhibited the progression of the cell cycle from the G1 to the S phase	[114]
20	Nagilactone E	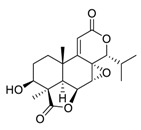	Activated the c-Jun N-terminal kinases, increased the phosphorylation, and promoted the localization of c-Jun in the nucleus	[115,116]
21	8-Oxo-epiberberine	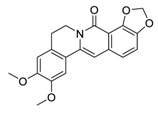	Inhibited TGF-β1-induced epithelial-mesenchymal transition (EMT) possibly by interfering with Smad3	[117]
22	Parthenolide	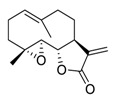	Reduced the phosphorylation of EGFR and downstream signaling pathways mitogen-activated protein kinase (MAPK)/ERK, inhibited PI3K/Akt/FoxO3α signaling	[118,119,120]
23	PDB-1	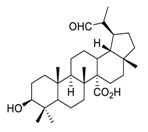	Suppressed lung cancer cell migration and invasion via FAK/Src and MAPK signaling pathways	[121]
24	Polyphyllin I	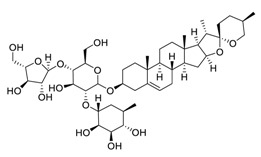	Induced autophagy by activating AMPK and then inhibited mTOR signaling, promoted apoptosis, modulated the PI3K/Akt signaling	[122,123]
25	Quercetin	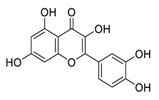	Inhibited proliferation and induced apoptosis	[124]
26	Silibinin	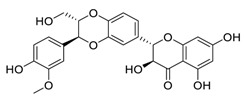	Inhibited cell proliferation, migration, invasion, and EMT expression	[125]
27	Sinomenine	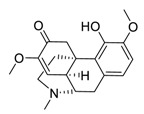	Downregulated expression of MMPs and miR-21, suppressed α7 nicotinic acetylcholine receptors expression	[126,127,128]
28	Toxicarioside O	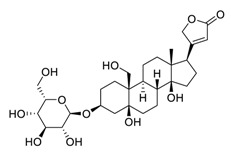	Decreased the expression of trophoblast cell surface antigen 2, resulting in inhibition of the PI3K/Akt pathway and EMT program	[129]
29	Vincamine	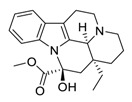	Interaction with the apoptotic protein caspase-3	[130]
30	Xanthohumol	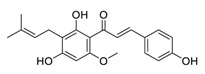	Suppressed ERK1/2 signaling and reduced the protein levels of FOS-related antigen 1, decreased the mRNA level of cyclin D1	[131]

## Data Availability

Not applicable.

## Abbreviations

ACE2	angiotensin-converting enzyme 2
ACE2+	overexpression of ACE2
AKT	protein kinase B
ATP	adenosine triphosphate
β-CD	β-cyclodextrin
3CLpro	3C-Like protease
COVID-19	coronavirus disease 2019
cryo-EM	cryo-electron microscopy
EBOV	Ebola virus
EC50	half-maximal effective concentration
EGFR	epidermal growth factor receptor
EMT	epithelial-mesenchymal transition
ERK	extracellular signal-regulated kinase
FDA	US Food and Drug Administration
GSH	glutathione
HA	hyaluronic acid
HCoV-229E	human coronavirus 229E
HCPNs	curcumin and cisplatin nanoparticles
HIV-1	human immunodeficiency virus type 1
IC50	half-maximal inhibitory concentration
MAPK	mitogen-activated protein kinase
Mcl-1	myeloid cell leukemia sequence-1
MERS-CoV	Middle East respiratory syndrome coronavirus
MMPs	matrix-degrading metalloproteinases
Mpro	main protease
mTOR	mammalian target of rapamycin
NSCLC	non-small cell lung cancer
PDB	Protein Data Bank
PD-L1	programmed death ligand-1
PI3K	phosphoinositide 3-kinase
PLpro	papain-like protease
pSARS-CoV-2	SARS-CoV-2 pseudovirus
QFPD	Qingfei Paidu decoction
RdRp	RNA-dependent RNA polymerase
ROS	reactive oxygen species
S protein	spike protein
SARS-CoV	severe acute respiratory syndrome coronavirus
SARS-CoV-2	severe acute respiratory syndrome coronavirus 2
SHL	Shuanghuanglian oral liquid or injection
SI	selectivity index
TMPRSS2	transmembrane protease serine 2
